# A live attenuated *Salmonella* Enteritidis secreting detoxified heat labile toxin enhances mucosal immunity and confers protection against wild-type challenge in chickens

**DOI:** 10.1186/s13567-016-0348-7

**Published:** 2016-06-04

**Authors:** Jonathan Lalsiamthara, Nitin Machindra Kamble, John Hwa Lee

**Affiliations:** Department of Bioactive Material Sciences and Department of Veterinary Public Health, College of Veterinary Medicine, Chonbuk National University, Iksan Campus, Iksan, 570-752 Republic of Korea

## Abstract

A live attenuated *Salmonella* Enteritidis (SE) capable of constitutively secreting detoxified double mutant *Escherichia coli* heat labile toxin (dmLT) was developed. The biologically adjuvanted strain was generated via transformation of a highly immunogenic SE JOL1087 with a plasmid encoding dmLT gene cassette; the resultant strain was designated JOL1641. A balanced-lethal host-vector system stably maintained the plasmid via auxotrophic host complementation with a plasmid encoded aspartate semialdehyde dehydrogenase (*asd*) gene. Characterization by western blot assay revealed the dmLT subunit proteins in culture supernatants of JOL1641. For the investigation of adjuvanticity and protective efficacy, chickens were immunized via oral or intramuscular routes with PBS, JOL1087 and JOL1641. Birds immunized with JOL1641 showed significant (*P* ≤ 0.05) increases in intestinal SIgA production at the 1^st^ and 2^nd^ weeks post-immunization via oral and intramuscular routes, respectively. Interestingly, while both strains showed significant splenic protection via intramuscular immunization, JOL1641 outperformed JOL1087 upon oral immunization. Oral immunization of birds with JOL1641 significantly reduced splenic bacterial counts. The reduction in bacterial counts may be correlated with an adjuvant effect of dmLT that increases SIgA secretion in the intestines of immunized birds. The inclusion of detoxified dmLT in the strain did not cause adverse reactions to birds, nor did it extend the period of bacterial fecal shedding. In conclusion, we report here that dmLT could be biologically incorporated in the secretion system of a live attenuated *Salmonella*-based vaccine, and that this construction is safe and could enhance mucosal immunity, and protect immunized birds against wild-type challenge.

## Introduction

*Salmonella enterica* serovar Enteritidis is one of the most frequently isolated bacteria from human infections worldwide [1]. Foodborne salmonellosis is widespread in developing and developed countries, resulting in approximated 155 000 deaths every year [2]. Eggs, meat and meat products are the most common transmission vehicles of *Salmonella* infections [3]. Due to the ubiquitous presence and rapid spread of *Salmonellae* in poultry premises, enforcing control measures can be expensive and still does not ensure complete elimination of the organism. Control strategies, such as culling, antibiotic interventions, and *Salmonella*-free feed approaches, are being deployed with varying success to control the *Salmonella* transmission cycle [4]. In particular, poultry vaccination is the suggested ideal strategy for controlling *Salmonella* Enteritidis infections on poultry farms and thereby reducing food contamination [5, 6].

The heat-labile enterotoxin (LT) of *E. coli* is a potent oral adjuvant boosting both the humoral and cellular immune responses when co-administered with antigens. However, it also induces secretory diarrhea, even at low doses [7, 8]. It is composed of a monomeric A subunit and pentameric B subunits [9]. The usefulness of native LT as an adjuvant is eclipsed by its toxicity. Amino acid substitutions have been introduced into the native LT to generate active but non-toxic mutant protein adjuvants. The first such example was the mutant labile toxin, mLT or LT(R192G), in which the amino acid glycine was substituted for arginine in the A-subunit, thereby preventing enzymatic cleavage [10]. mLT showed reduced toxicity [11] and maintained adjuvanticity in vitro and in animal studies, inducing a balanced Th1/Th2 cytokine and antibody subclass profile equivalent to native LT [12–14]. However, at a higher dosage (100 µg of mLT), human subjects showed mild to moderate diarrhea [15]. To alleviate this problem, an additional mutation was added to further detoxify the toxin and thus generate the double mutant, LT (R192G/L211A), or dmLT [16]. Detoxified dmLT has reduced cyclic AMP activation and exhibited no enterotoxicity, but most importantly, it retained the ability to function as an oral mucosal adjuvant. LT and its variants can enhance immune responses to whole cell vaccines against enterotoxigenic *E. coli*, *Streptococcus pneumonia* and *Helicobacter pylori* in different mouse models [17, 18].

Several studies have utilized conjugation of purified recombinant dmLT protein with candidate antigens for immunological studies, dose optimizations, and vaccine development [10, 19, 20]. However, based on our previous studies with adjuvanted vaccine strains, we perceived that the use of strains inherently secreting [21] or displaying [22] adjuvant molecules would be more practical for bulk production and may be more convenient for field deployments. In this study, to improve the vaccine-biological adjuvant system and to surpass the multistep processing of dmLT-conjugations, we investigated the use of a highly immunogenic live attenuated SE strain that secretes dmLT adjuvant molecules constitutively as a vaccine candidate. The protective efficacy against virulent challenge, immune responses, and safety as a vaccine candidate, were assessed in a chicken model.

## Materials and methods

### Experimental birds, bacterial strains and plasmids

Male white leghorn chickens were used to examine the protective efficacy, induction of immune response, and safety. All experimental work involving birds was approved (CBU 2014-1-0038) by the Chonbuk National University Animal Ethics Committee, in accordance with the guidelines of the Korean Council on Animal Care. The bacterial strains, plasmids, and primers used are listed in Table 1. All *E. coli* and *Salmonella* Enteritidis strains were grown in Luria–Bertani (LB) broth. For bacterial counting, *Salmonella* strains were grown on brilliant green agar (BGA) at 37 °C, for enrichment and recovery they were grown in Rappaport–Vassiliadis broth at 42 °C. Fifty µg/mL of diaminopimelic acid supplement was added to media for growing *asd* gene-deleted strains.

**Table 1:** Bacterial strains and plasmids used in this study

Strain/plasmid	Description	Reference
*Salmonella* Enteritidis		
JOL1182	Wild type isolate from chicken, challenge strain	Lab stock
JOL860	Wild type isolate from chicken for antigen preparations	Lab stock
JOL1087	Δ*lon*Δ*cpxR*Δ*asd,* used as base vaccine strain	[21]
JOL1641	JOL1087 containing pJHL65-dmLT	This study
Plasmids		
pJHL65	*asd* + vector, pBR ori, b-lactamase signal sequence-based periplasmic secretion plasmid, 6xHis, high copy number	[34]
pJHL65-dmLT	pJHL65 containing dmLT constitutively express under Ptrc promoter, secreted under *bla* secretory system	This study

### Construction of plasmids harboring dmLT

**Figure 1:**
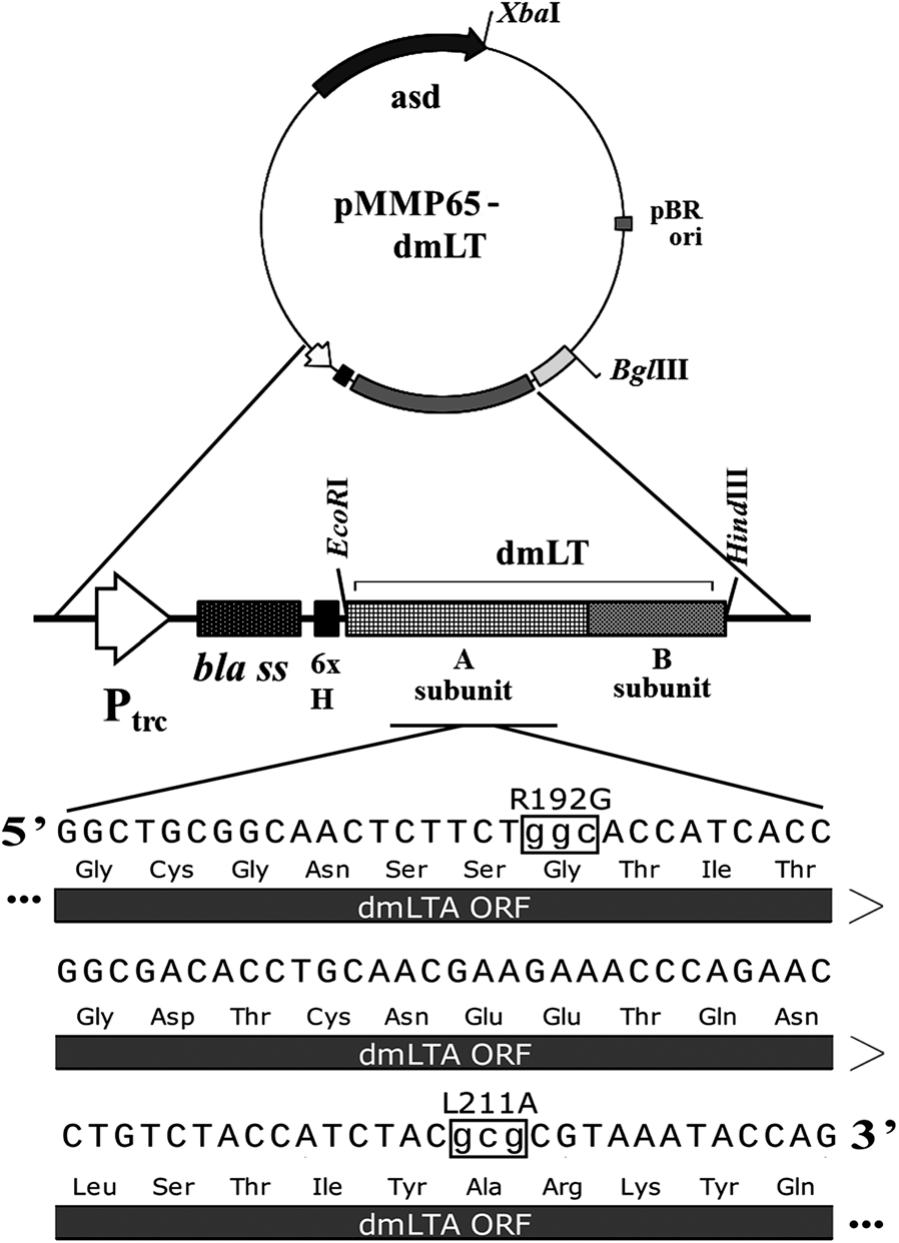
**Components of pJHL65-dmLT.** The SE *asd* + plasmid with ori pBR (pJHL65) harboring a constitutive expression system under which the dmLT cassette was expressed along with a fused *bla* secretion signal sequence. Lower case DNA sequence (inside the box) depicts codon substitution and corresponding non-synonymous amino acid substitutions, R192G and L211A.

The codon-optimized dmLT gene incorporating the desired nucleotide mutations was chemically synthesized (Bioneer, South Korea). It consisted of the open reading frame (ORF) of the LTA subunit and an overlapping ORF of the LTB subunit (Figure 1). The desired amino acid substitutions in the LTA subunit were glycine instead of arginine and alanine instead of leucine at positions 192 and 211, respectively [16]. The synthesized gene cassette was enzymatically released via the incorporated *EcoR*I and *Hind*III restriction sites. The released fragment was then cloned into the based vector pJHL65. The constructed plasmid consisted of the cloned *asd* gene that assisted plasmid-host complementation, the dmLT cassette fused with a *bla* secretion signal and a 6xHis tag sequence. The fused cassette was constitutively expressed under the Ptrc promoter. The plasmid was then used to transform JOL1084 (Δ*lon*Δ*cpxr*Δ*asd*) via electroporation. The resulting strain was designated JOL1641 (Table 1). For comparative studies, JOL1084 was transformed with *asd* + pMMP65 without the dmLT cassette.

### Immunoblot confirmation of dmLT expression in culture supernatants

dmLT subunit expression was confirmed from the culture supernatant of JOL1641 using a previously described protocol with minor modifications [21]. Briefly, 200-mL supernatants from broth cultures grown at 37 °C (OD_600_ 0.8) were recovered following centrifugation at 3380 *g* for 15 min. The supernatant containing the secreted protein was filtered through a 0.22-µm pore-size filter and precipitated with chilled 20% trichloroacetic acid overnight. The precipitated proteins were pelleted at 30 000 *g* for 20 min, washed with acetone and resuspended in PBS. After heating at 96 °C for 5 min, the proteins were separated using 15 and 18% SDS-PAGE gels for LTA and LTB, respectively. The separated proteins were blot transferred to polyvinylidene fluoride (PVDF) membranes (Millipore, USA) and overnight blocking was performed in 5% skim milk. The LTA subunit was detected via the incorporated histidine tags, using primary mouse IgG1 anti-His-tag (Penta-His™, Life Technologies, Eugene, OR, USA) and secondary anti-mouse IgG1 antibody-HRPO conjugate (Sigma-Aldrich, USA) antibodies. The LTB subunit was detected using a lab-generated hyperimmune serum primary antibody (rabbit anti-LTB) and a secondary anti-rabbit IgG antibody-HRPO conjugate (Sigma-Aldrich). Reactive bands were developed using the West-One™ Western Blot Detection System (iNTRON, KOR) and bands were visualized using a KODAK Image Station (Kodak, New Haven, CT, USA).

### GM1 ganglioside binding assay

GM1 based functional assay was conducted to validate the presence of dmLT in culture supernatants. GM1-based sandwich ELISA was performed based on a previously reported protocol with minor modifications [22]. The modifications were application of dmLT protein and use of anti-LTA antibody. Briefly, 5 µg/mL of purified ganglioside GM1 from bovine brain (Santa Cruz Biotechnology, USA) was used for coating the ELISA plate, and the remaining binding sites were blocked by incubating the plates with 200 μL of 5% skimmed milk. After washing three times with PBST, precipitated supernatants of JOL1640 was added and incubated at 37 °C for 2 h. The unbound sites were blocked with 200 μL of 5% skimmed milk. A 1:5000 dilution of anti-LTA rabbit serum was added to the wells and then incubated at 37 °C for 1 h. After washing five times with PBST, the plates were incubated with a 1:10 000 dilution of HRP-conjugated goat anti-rabbit IgG secondary antibody. The activity of bound HRP was measured using OPD (Sigma-Aldrich, St. Louis, MO, USA). A negative binding control involving all the steps was also carried out in parallel, except that GM1 coating was replaced with 5% skimmed milk.

### Immunization and challenge of chickens

Fifty white leghorn chickens were divided equally into five groups (*n* = 10) and were immunized orally or intramuscularly with PBS (non-immunized control), JOL1087 (parental strain) and JOL1641 (SE-dmLT). All chickens were immunized at 4 weeks of age. The immunization regime consisted of 1 × 10^8^ cells in 200 µL PBS for the oral route and 1 × 10^7^ cells in 200 µL PBS for the intramuscular route (Table 2). The birds were then simultaneously utilized for safety assessment, immunological and protective efficacy studies. All bird groups were challenged during the 5^th^ week post-vaccination with 1 × 10^9^ CFU in 200 µL using a virulent *Salmonella* Enteritidis strain (JOL1182) administered via the oral route.

**Table 2:** Immunization of hens with the *Salmonella* Enteritidis strains and challenge

Group (*n* = 10)	Constructed strain	Route	Dosage^a^	Challenge^b^
A	PBS control	Oral	200 µL sterile PBS	
				JOL1182 1 × 10^9^ cells/200 µL PBS
B	JOL1087	Oral	1 × 10^8^	
		Intramuscular	1 × 10^7^	
C	JOL1641	Oral	1 × 10^8^	
		Intramuscular	1 × 10^7^	

^a^Dose for immunization expressed as number of Salmonella Enteritidis cells/200 µL of PBS.

^b^Chickens were challenged at the 9^th^ week of age via oral route. Chickens were euthanized at 7 and 14 days post-challenge.

### Safety and fecal shedding of vaccine strains

The safety qualities of the strains were investigated through regular monitoring of the birds until euthanization. The investigated parameters included anorexia, depression, and diarrhea. The presence of the vaccine strains in fecal samples was monitored 3, 7, 14 and 21 days post-immunization. Fecal samples were processed using a protocol described in detail elsewhere [21]. Birds were kept in clean disinfected buckets prior to fecal sampling. The fecal samples were collected and resuspended at a ratio of 1:10 in buffered peptone water. After brief pelleting, various dilutions of the supernatant were plated directly on BGA plates. Simultaneously, 1 mL of the supernatant was also enriched in 4 mL RV broth and incubated 48 h at 42 °C. A loop of the enrichment broth was streaked onto BGA and incubated at 37 °C for 16 h. *Salmonella*-like colonies that appeared after plating or streaking on BGA were further confirmed using SE-specific [23] and candidate strains specific primers.

### Protective efficacy and bacterial recovery from infected organs

To evaluate the protective efficacy of the SE-dmLT strains, immunized birds were challenged with the SE virulent strain JOL1182 as described above. The protective efficacy of the dmLT-secreting strain JOL1641 was compared with those of the non-immunized control and the parental strain JOL1087. The birds were classified into Groups A, B and C corresponding to inoculation with PBS only and strains JOL1087 and JOL1641 respectively. The challenge experiment was carried out according to the protocol described previously with minor modifications [21]. At days 7 and 14 post-challenge, the birds were euthanized, and organ bacterial recovery and post-mortem examination were performed. To determine bacterial loads, samples of the liver, spleen, and cecum were weighed and then homogenized in 2 mL buffered peptone water. Hundred µL of the homogenate sample was plated on BGA for direct culture. The resulting colonies were counted after incubation at 37 °C for 16 h. In parallel, the remaining 1 mL homogenate was enriched in 4 mL of RV broth and incubated 48 h at 42 °C. A loop of the enrichment broth was streaked onto BGA and incubated at 37 °C for 16 h. *Salmonella*-like colonies were further confirmed using specific PCR primers. The number of bacterial colonies obtained via direct culturing were determined and expressed as the mean log_10_ CFU/g of samples. For the purposes of statistical analysis, a sample that was positive only after enrichment was rated as log_10_ = 1.0. A sample that was negative after enrichment was assigned as log_10_ = 0 [24].

### Assessment of systemic and mucosal humoral immune responses

To measure anti-SE-specific antibodies, blood, and intestinal lavage samples were collected at weekly intervals for 5 weeks post-immunization. Heparinized peripheral blood was used to recover plasma samples to determine systemic immunoglobulin G (IgG) concentrations. Intestinal lavage samples were collected to determine secretory IgA (SIgA) concentrations, as per the protocol described elsewhere [25]. Indirect ELISA was performed for IgG and SIgA with an outer membrane protein (OMP) fraction extracted from wild-type JOL860, following a previously described protocol with minor modifications [26]. Changes included 40 min centrifugation at 20 000* g* of sonicated pellet and final harvesting at 130 000* g* for 1 h at 4 °C. IgG and SIgA samples were diluted with PBS at ratios of 1:100 and 1:10, respectively. Each sample was tested in duplicate, and the mean OD_492_ was calculated for each time point and compared to that of control samples.

### Statistical analysis

Statistical analyses were applied wherever applicable. Analysis of variance (ANOVA) was used to analyze the differences among the group means; Tukey’s HSD post hoc analysis was further used to differentiate within groups. Differences were considered statistically significant at a *P* value of ≤0.05. In order to achieve a better statistical fit, bacterial enumeration log counts “x” at day 14 post-challenge (orally immunized groups) were mathematically transformed using the equation *y* = (*x* + 1).

## Results

### Construction and validation of dmLT

**Figure 2:**
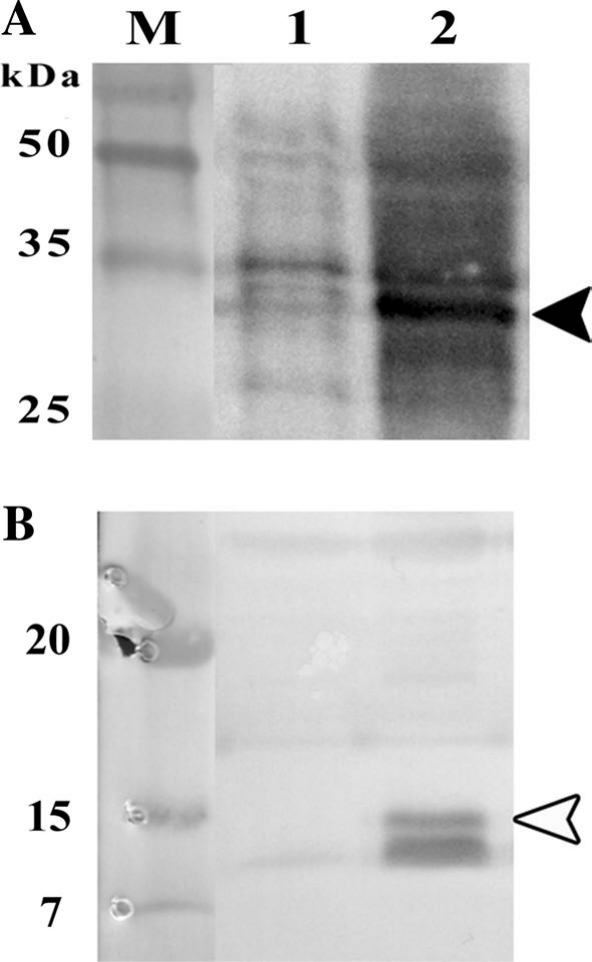
**Western blot validation of dmLT from JOL1641.** Lane M, prestained protein marker; lane 1, supernatant from the blank vector control; lane 2, JOL1641. **A** Immunoblot analysis of the LTA subunit, black arrow head poinitng the ~32-kDa reactive protein band. **B** Immunoblot analysis of the LTB subunit, white arrow head indicating the ~12-kDa reactive protein band.

The synthesized dmLT cassette containing suitable nucleotide mutations was successfully cloned inside the backbone vector pJHL65 (Figure 1). The expression and secretion of dmLT in JOL1641 were validated using precipitated secretory protein harvested from a JOL1641culture. dmLT subunit A and subunit B were detected by using anti-His tag antibody and anti-LTB polyclonal antibody, respectively. The total size of LTA (29 kDa) fused with 6xHis and a signal peptide (3 kDa) was approximately 32 kDa. The expected band sizes of 32 and 12 kDa corresponding to monomeric A and B subunits, respectively, were observed on the western blots (Figure 2). Holotoxin formation was confirmed based on LTB binding to GM1 ganglioside receptor. Mean OD ± SEM values of 0.3608 ± 0.0117 and 0.1353 ± 0.0078 were observed for GM1-dmLT and non-GM1-dmLT wells. Minimal OD was observed with GM1 negative wells due to the absence of toxin-receptor complex. Hence, no or minimal primary and conjugated-secondary antibodies were available for generating colour reaction.

### Safety and fecal bacterial shedding

No adverse effects on the general health conditions of the immunized birds were observed. Native heat labile toxin has the tendency to evoke severe diarrhea; however, with the suitable mutations in the LTA subunit, JOL1641 did not induce diarrhea among the immunized birds. Furthermore, upon postmortem examination, no conspicuous pathological lesions, such as organ enlargement, infarctions or necroses, were observed. The expression of detoxified toxin in JOL1641 did not prolong fecal shedding in the birds. The shedding pattern of JOL1641-immunized birds in Group C was comparable to that of Group B JOL1087-immunized birds (Table 3).

**Table 3:** Faecal shedding of constructed strains post immunization

Group	Constructed strain	Route	Day			
			3	7	14	21
A	PBS control	Oral	0/6^a^	0/6	0/6	0/6
B	JOL1087	Oral	4/6	3/6	0/6	0/6
		Intramuscular	2/6	0/6	0/6	0/6
C	JOL1641	Oral	4/6	2/6	0/6	0/6
		Intramuscular	0/6	0/6	0/6	0/6

^a^Number of birds positive for faecal shedding/number of birds screened.

### Systemic and mucosal humoral immune responses

**Figure 3:**
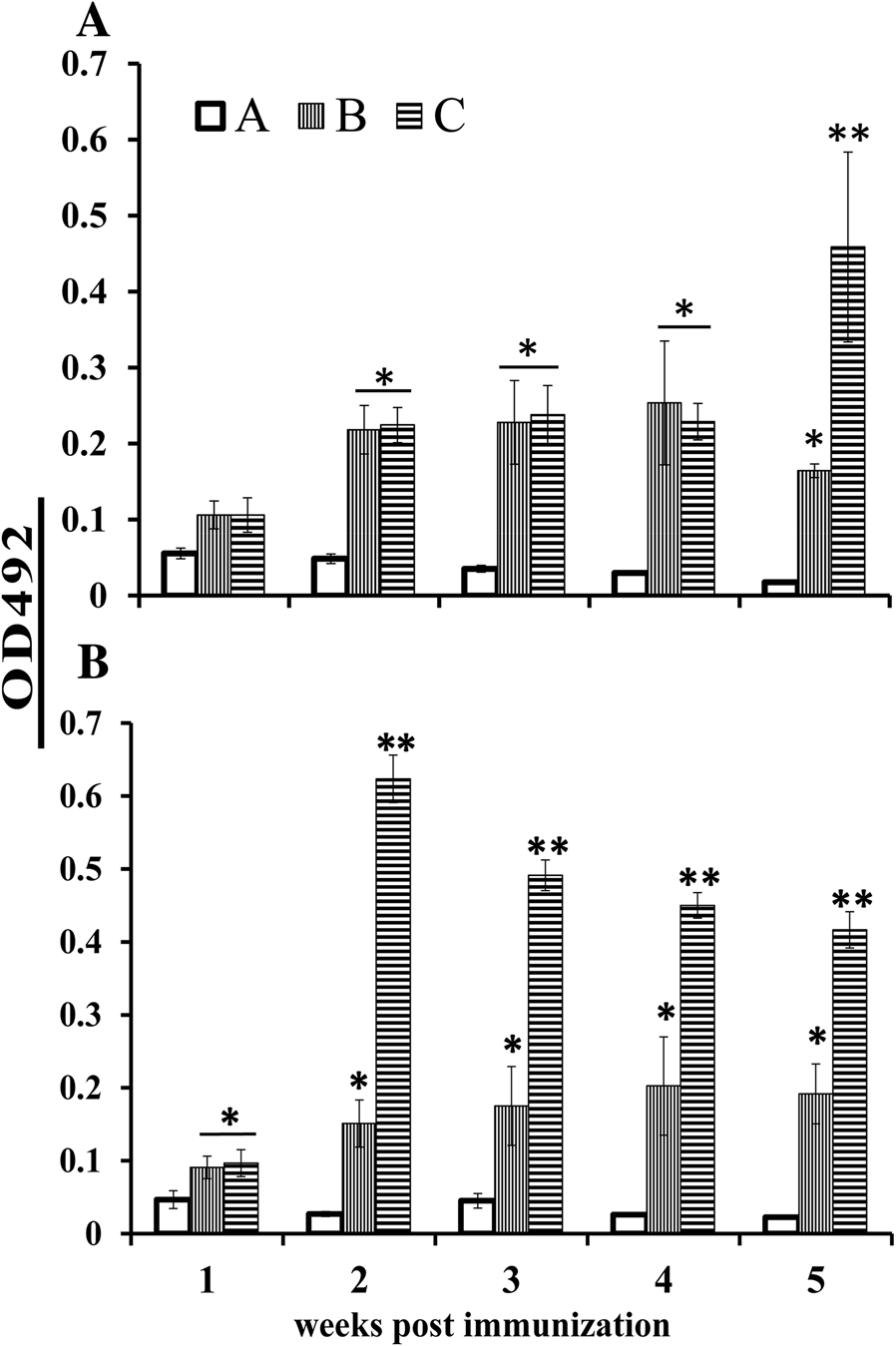
**Relative plasma IgG levels in immunized birds.** IgG antibodies produced against the SE outer membrane protein antigens were measured using indirect ELISA. Bird groups were inoculated with: A, PBS control; B, JOL1087 and C, JOL1641. **A** Groups inoculated via the oral route. Immunized birds showed significant differences in their IgG levels as compared to the control group. This significant difference was observed at the 2^nd^ week post-immunization. **B** Groups inoculated via the intramuscular route. Birds in Group C showed prominent increase in the IgG levels that declined gradually. As compared to the non-immunized group, the immunized groups showed significant differences at all sampling time points, except during the first week. *Significant difference compared to the control group (*P* ≤ 0.05), **Significant difference between immunized groups (*P* ≤ 0.05).

**Figure 4:**
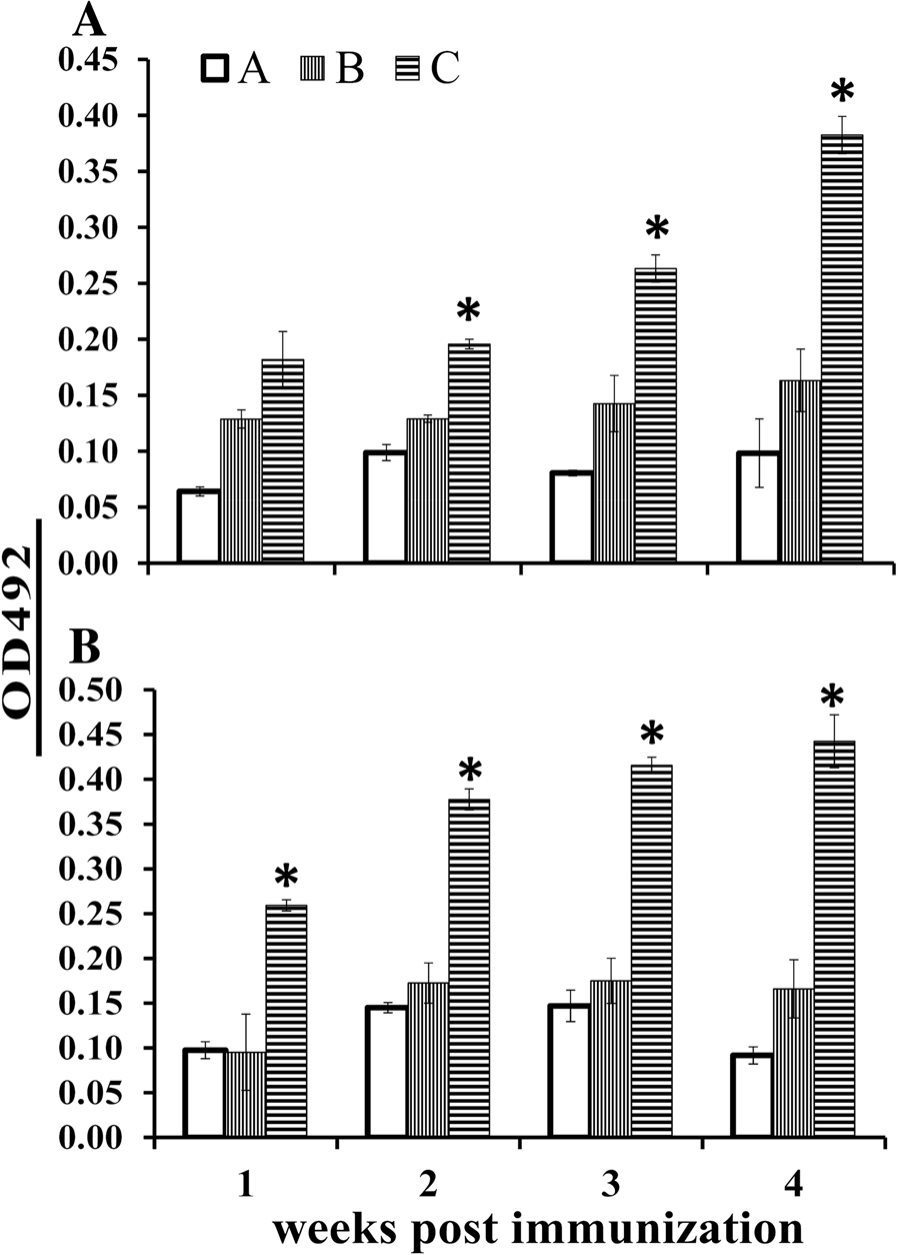
**Relative intestinal SIgA levels in immunized birds.** SIgA antibodies produced against the SE outer membrane protein antigens were measured using indirect ELISA. Bird groups were inoculated with: A, PBS control; B, JOL1087 and C, JOL1641. **A** Bird groups immunized via oral route. At the second week post-immunization, Group C birds showed significant rises in SIgA levels as compared to the other groups. **B** Bird groups immunized via the intramuscular route. At the first week post-immunization, Group C birds showed significant increases in SIgA. Birds in Group C showed consistent increases in SIgA levels 2, 3 and 4 weeks post-immunization. *Significant difference compared to the other groups (*P* ≤ 0.05).

The systemic and mucosal humoral immune responses elicited by JOL1641 were investigated by measuring the levels of plasma IgG and intestinal lavage SIgA from samples obtained from immunized and non-immunized chickens. All immunized birds elicited strong antibody production against the SE specific outer-membrane protein antigens. Bird groups immunized via the oral route showed a significant (*P* ≤ 0.05) rise in IgG antibody titers as early as the second week post-immunization, compared to the non-immunized group (Figure 3A). Group B birds showed a declining level of IgG beyond the 4^th^ week post-immunization while Group C birds showed significantly (*P* ≤ 0.05) higher levels of IgG at the 5^th^ week post-immunization. Bird groups immunized via the intramuscular route showed significant increases in IgG levels from the first week post-immunization as compared to the control group (Figure 3B). SE-dmLT adjuvant inoculated birds (Group C) showed an abrupt rise in IgG levels by the 2^nd^ week, which gradually decreased towards the 5^th^ week post-immunization; the observed levels were significantly higher than those of Groups A and B. To evaluate the role of SE-dmLT in inducing mucosal immunity, the levels of intestinal SIgA were measured. The SIgA levels of orally immunized birds showed significant differences as compared to control non-immunized birds (*P* ≤ 0.05). At the first and 2^nd^ week post oral immunization, the SIgA levels were relatively similar between the groups; by the second week, Group C birds showed significant differences in SIgA levels as compared to Group B birds (Figure 4A). Interestingly, Group C birds had significantly (*P* ≤ 0.05) higher concentrations of SIgA at all sampling time points compared to Groups A and B post intramuscular immunization (Figure 4B).

### Challenge and protection study

**Figure 5:**
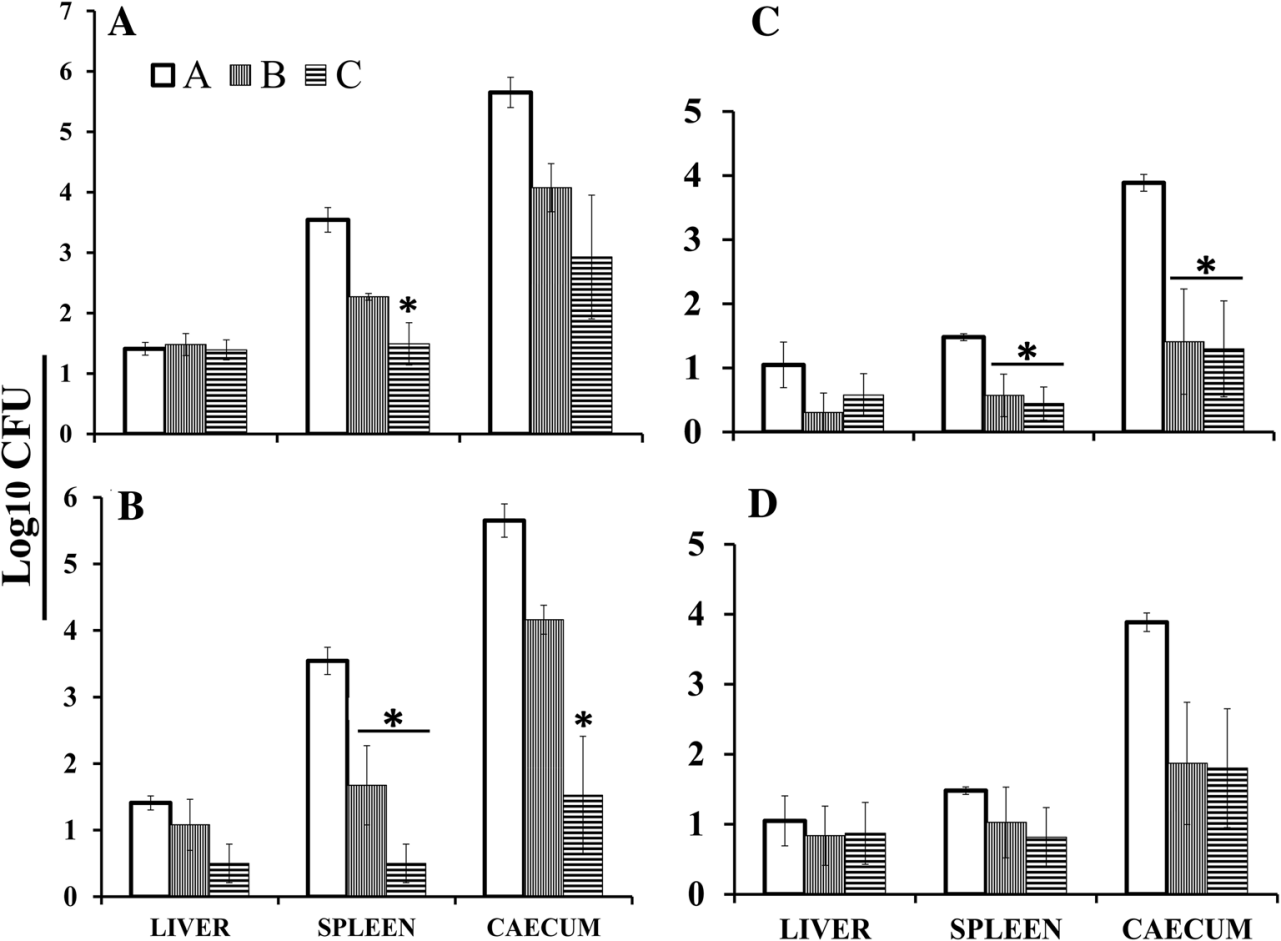
**Recovery of challenge strain from internal organs of chickens**. Enumeration of wild type bacterial load was performed in liver, spleen, and cecum of the bird after virulent wild type challenge. (**A**) Log10 organ bacterial counts of bird groups immunized orally, at the 7^th^ day post-challenge. Spleens from Group C showed significant reduction in challenge bacterial load as compared to Group A and B (*P* ≤ 0.05) birds. (**B**) Log10 organ bacterial counts of bird groups immunized intramuscularly, at the 7^th^ day post-challenge. The splenic bacterial loads were significantly lower in Group B and C as compared to Group A (*P* ≤ 0.05). The caecal bacterial load was significantly lower in Group C, birds immunized with adjuvanted strain (*P* ≤ 0.05). (**C**) Log10 organ bacterial counts of bird groups immunized orally, at the 14^th^ day post-challenge. Spleens and ceca of Group B and C showed significant decreased in bacterial loads as compared to the control group A (*P* ≤ 0.05). (**D**) Log10 organ bacterial counts of bird groups immunized intramuscularly, at the 14^th^ day post-challenge. In Group B and C, the number of birds determined to be completely negative of the challenge strain were 4 of 10.

The protective efficacy of the strain was investigated by immunizing 4-week-old birds. The immunized birds were challenged with wild-type JOL1182 in the 5^th^ week post-immunization. Birds were euthanized on days 7 and 14 post-challenge, the gross appearance of postmortem organs was examined and the wild type bacterial load was counted in selected organs including, liver, spleen, and cecum. Apart from mild splenomegaly, no severe pathological lesions were observed in the internal organs of the immunized birds. On the 7^th^ day post-challenge, birds immunized orally did not show signs of having significantly cleared the infection from their livers any faster than the control non-immunized birds (Figure 5A). The splenic bacterial loads were significantly lower in Group C birds (*P* ≤ 0.05). On the 7^th^ day post-challenge, bird groups immunized via the intramuscular route showed significantly lower bacterial loads compared to control birds (*P* ≤ 0.05; Figure 5B). The splenic bacterial counts were significantly lower in both Group B and C birds (*P* ≤ 0.05). The cecal bacterial counts were significantly lower in Group C birds (*P* ≤ 0.01) compared to Group A and B birds. On the 14^th^ day post-challenge, splenic and caecal counts were significantly lower in bird groups immunized orally (*P* ≤ 0.05; Figure 5C). On day 14 post-challenge, the log bacterial counts were reduced among the bird groups immunized intramuscularly, compared to the non-immunized group of birds (Figure 5D). Of the ten birds in both Groups B and C, four were found to be negative for the SE challenge strain, while all ten birds in the Group A control group were found to be positive.

## Discussion

Upon infection via the oral route, *Salmonella* spreads rapidly, crossing the intestinal mucosa and invading the spleen and liver. The ability of *Salmonella* to evade killing by phagocytic cells and its pan tropism towards a variety of non-phagocytic cells leads to massive bacterial replication, resulting in high bacterial loads and systemic *Salmonella* infection [27]. It would be pragmatic to halt the invading *Salmonella* organisms before they penetrate their replication niche. Essentially, SIgA antibodies present in the intestinal mucus act as an important immunological barrier, preventing adherence and subsequent invasion of the intestinal lining by *Salmonella*. The present study investigated the effectiveness of the complete molecule of detoxified *E. coli* labile toxin dmLT in enhancing immunity against wild type *Salmonella* Enteritidis infection. The double mutant labile toxin gene was fused with the *bla* secretion signal sequence and was expressed under the constitutive promoter Ptrc. Furthermore, the stability of the plasmid was maintained by host-plasmid complementation using the *asd* gene. *Salmonella asd* gene knock-out strains transformed with this plasmid were stable and constitutively produced dmLT proteins, which are directed to the bacterial periplasmic space and then secreted. In order to achieve a strong protective ability and immunogenic properties, the dmLT adjuvanted strain JOL1641 was developed based on the highly immunogenic *lon* and *cprxR* gene-deleted SE strain (JOL1087). Deletion of *lon* and *cprxR* renders the organism attenuated by impairing intracellular replication while increasing its immunogenicity by up-regulating adhesion and invasion [28–30]. These properties of JOL1087 added additional suitable qualities to the vaccine candidate as evidenced by plasma IgG levels (Figure 3), intestinal lavage SIgA antibody levels (Figure 4) and protection (Figure 5).

Expression of dmLT was confirmed by immunoblot assay, which revealed the presence of the LTA and LTB proteins in the secretions of JOL1641 (Figure 2). As reported earlier, the double mutations in the gene for *E. coli* labile toxin eLT resulted in inhibition of proteolytic cleavage of LT-A into A1 and A2. It exhibited reduced enzymatic activity and no detectable toxicity either in vitro or in vivo [16]. Our observations confirmed that the incorporation of dmLT in the strain did not cause any additional undesirable effects, like general illness, diarrhea or prolonged fecal shedding (Table 3). In addition, no immunization-induced pathological lesions were observed in the internal organs upon postmortem examination.

Native toxins such as cholera and labile toxins and their variant toxoids can enhance both humoral and cell-mediated immunity [17, 31, 32]. Humoral immunity plays an important role in the early stages of infection, during which extracellular *Salmonella* Enteritidis is opsonized by antibodies, thereby preventing cell penetration [33]. Systemic antibodies opsonize *Salmonella* Enteritidis and thus enhance receptor-mediated uptake by macrophages. Our data revealed that the candidate strain was highly immunogenic when administered either orally or intramuscularly. The strain was capable of inducing strong anti-*Salmonella* specific systemic IgG, and the levels were significantly different from those of control birds. We observed a significant induction effect of mucosal adjuvant on intestinal SIgA production by the 2^nd^ week post-immunization upon oral administration among Group C birds (Figure 4A). Interestingly, SIgA production in the intestinal lavages was significantly higher during the 1^st^ week post-immunization via the intramuscular route among Group C birds; this significant rise was observed 1 week earlier compared to the oral route of immunization. Overall, our data indicated that oral and intramuscular administration of SE-dmLT resulted in a significant elevation of SIgA production, compared to the non-adjuvanted SE strain.

*Salmonella* live attenuated vaccines are known to induce a strong immune response [6]. In order to determine the effects of the vaccines in the early stages of infection, enumeration of the challenge strain log count was carried out on the 7^th^ day post-challenge. Correlations were drawn between the bacterial counts and the high SIgA titers observed in Group C via oral administration (Figure 4A). The splenic log count was significantly reduced in the adjuvanted group (Figure 5A). The increase in SIgA production induced by dmLT may have helped to neutralize the challenge bacteria and thus lessen splenic localization. As far as oral immunization is concerned, we speculate that the inclusion of dmLT improved the candidate vaccine strain by eliciting higher levels of intestinal SIgA production and, thereby may have prevented splenic colonization sooner than in the non-dmLT counterpart birds. Furthermore, all birds immunized via the intramuscular route showed a similar pattern of significantly reduced bacterial counts, irrespective of adjuvant inclusion (Figure 5B). The ceca of immunized birds were also significantly protected relative to those of control non-immunized groups (*P* ≤ 0.05). The overall organ bacterial counts were lower on the 14^th^ day post-challenge than on the 7^th^ day (Figures 5C and D). Furthermore, the number of birds negative for the challenge strain was higher on the 14^th^ day post-challenge (Table 4).

**Table 4:** Isolation of challenge strain from internal organs of birds

Group	Constructed strain	Route	Day post challenge	Organ^a^		
				Liver	Spleen	Caecum
A	PBS control	PBS	7	10/10	10/10	10/10
B	JOL1087	Oral	7	10/10	10/10	10/10
		Intramuscular	7	8/10	8/10	10/10
C	JOL1641	Oral	7	8/10	8/10	10/10
		Intramuscular	7	5/10	5/10	6/10
B	JOL1087	Oral	14	3/10	5/10	5/10
		Intramuscular	14	6/10	6/10	6/10
C	JOL1641	Oral	14	5/10	5/10	5/10
		Intramuscular	14	6/10	4/10	6/10

^a^Number of organs positive of wild type SE/total number of organs examined.

Our data demonstrated that JOL1641 could significantly protect immunized birds from virulent wild-type challenge. The inclusion of adjuvant dmLT in the secretions of JOL1641 strain increased SIgA production in the intestines of immunized birds. These increased SIgA levels may correlate with better splenic protection during early infection. In conclusion, this study reports a unique vaccine development strategy involving a live attenuated vaccine and an intrinsically incorporated mucosal adjuvant. We also provided compelling data that this novel strain could be a new tool for poultry anti-*Salmonella* Enteritidis vaccination, especially as a means to boost the humoral immune response required for neutralization during the early phases of invading *Salmonellae* infection.
